# Characterizing the Increase in Artificial Intelligence Content Detection in Oncology Scientific Abstracts From 2021 to 2023

**DOI:** 10.1200/CCI.24.00077

**Published:** 2024-06-01

**Authors:** Frederick M. Howard, Anran Li, Mark F. Riffon, Elizabeth Garrett-Mayer, Alexander T. Pearson

**Affiliations:** ^1^ Section of Hematology/Oncology, Department of Medicine, The University of Chicago, Chicago, IL; ^2^ Center for Research and Analytics, American Society of Clinical Oncology, Alexandria, VA

## Abstract

**PURPOSE:**

Artificial intelligence (AI) models can generate scientific abstracts that are difficult to distinguish from the work of human authors. The use of AI in scientific writing and performance of AI detection tools are poorly characterized.

**METHODS:**

We extracted text from published scientific abstracts from the ASCO 2021-2023 Annual Meetings. Likelihood of AI content was evaluated by three detectors: GPTZero, Originality.ai, and Sapling. Optimal thresholds for AI content detection were selected using 100 abstracts from before 2020 as negative controls, and 100 produced by OpenAI's GPT-3 and GPT-4 models as positive controls. Logistic regression was used to evaluate the association of predicted AI content with submission year and abstract characteristics, and adjusted odds ratios (aORs) were computed.

**RESULTS:**

Fifteen thousand five hundred and fifty-three abstracts met inclusion criteria. Across detectors, abstracts submitted in 2023 were significantly more likely to contain AI content than those in 2021 (aOR range from 1.79 with Originality to 2.37 with Sapling). Online-only publication and lack of clinical trial number were consistently associated with AI content. With optimal thresholds, 99.5%, 96%, and 97% of GPT-3/4–generated abstracts were identified by GPTZero, Originality, and Sapling respectively, and no sampled abstracts from before 2020 were classified as AI generated by the GPTZero and Originality detectors. Correlation between detectors was low to moderate, with Spearman correlation coefficient ranging from 0.14 for Originality and Sapling to 0.47 for Sapling and GPTZero.

**CONCLUSION:**

There is an increasing signal of AI content in ASCO abstracts, coinciding with the growing popularity of generative AI models.

## Teaser

Analysis of 15,553 ASCO meeting abstracts with 3 AI detectors suggests rise in AI content in 2023.

## INTRODUCTION

Large language models (LLMs) are a form of deep neural networks, often with billions of tunable parameters, that are trained on a vast corpus of text to accurately reflect human language. ChatGPT is a LLM-based online chatbot from OpenAI, released November 2022, which drove unprecedented use in the months after its launch. Subsequently, LLMs such as ChatGPT have been widely applied throughout medical research to generate artificial intelligence (AI) for tasks such as responding to questions from patients on an online forum,^1^ passing medical licensing examinations,^2^ and summarizing clinical text.^3^ Even without specific training on oncology data sets, this new generation of tools can be applied to cancer-specific tasks such as identifying metastatic progression within computed tomography scan reports^4^ or precisely answering radiation oncology physics questions.^5^

**CONTEXT**

**Key Objective**
To describe the accuracy of three artificial intelligence (AI) content detectors and characterize the use of AI in generating text for scientific abstracts in ASCO Annual Meetings.
**Knowledge Generated**
All detectors achieved >95% sensitivity for AI content, with 1% or less false-positive rate, when tested on 100 abstracts before the advent of modern AI language tools paired with 100 abstracts created by GPT-3.5/GPT-4 models. Abstracts from 2023 were associated with a two-fold higher likelihood of AI content than abstracts from previous years, associated with online-only presentation and lack of a clinical trial number.
**Relevance *(J.L. Warner)***
Scientific meetings are one of the most important venues for conveying results including those with practice-changing implications. In the past several years, large language models (LLMs) have become pervasive and as such it is somewhat expected that they are being used to generate some portion of the scientific discourse. It will be important to recognize and monitor this LLM-generated content, given the potential issues with accuracy, originality, and trustworthiness.*
*Relevance section written by *JCO Clinical Cancer Informatics* Editor-in-Chief Jeremy L. Warner, MD, MS, FAMIA, FASCO.

Given the general medical knowledge encoded by LLMs, these models have been applied not only to answer scientific and medical questions, but also to produce scientific literature. Several studies have demonstrated that LLMs can produce convincing scientific articles—which drastically saves time in manuscript writing, but can produce fictitious references or citations.^6,7^ LLMs have also been applied to generate immediate feedback on research manuscripts, with one study reporting that the overlap between GPT-4 feedback and human reviewer feedback was comparable with the overlap between two human reviewers, although the model comments lacked in-depth critique.^8^ LLMs have also been found helpful for brainstorming initial research ideas, summarizing extensive literature, and performing preliminary analysis, with limitations such as lack of coherence or authenticity in composing complete sections in research paper drafts.^9^ The practical use of LLMs in scientific literature is far from theoretical—in a recent highly publicized example, a scientific journal published an article with the obvious AI-written text in the introduction—“Certainly, here is a possible introduction for your topic.”^10^ A survey of 1,600 researchers demonstrated that 25% are already using AI to assist in scientific writing, and this number is likely to grow with the development of more sophisticated LLM models and greater familiarity and ease of access to AI tools.^11^ LLMs will therefore have an indelible impact on all aspects of scientific writing from abstract preparation, manuscript drafting, grant proposals, and peer review.

Usage of AI tools such as LLMs in scientific writing remains controversial because of the risk of hallucinated (ie, plausible sounding but false) or inaccurate text generation, and AI models cannot be held accountable for generated content. Prestigious journals such as *Science* and *Nature* have stated that no generative AI can be listed as an author and have either prohibited usage of such tools^12^ or required researchers to disclose how they are used.^13^ However, these restrictions remain difficult to enforce, as outside of clearly fabricated references or common AI response phrases, detection of AI content remains challenging. A study comparing 50 generative pretrained transformer (GPT)-generated abstracts and human-written abstracts found that AI-generated texts were easily identifiable by AI content detectors, whereas blinded human reviewers inconsistently identified the AI-written abstracts.^14^ With the advent of more accurate LLMs, an increasing number of AI content detection tools are available, with varying accuracy in detecting AI-written scientific text.^15,16^

Given the inaccuracy in human identification of AI text, AI content detectors may be an invaluable screening tool for scientific abstracts, but the comparative accuracy and performance characteristics of detectors is not known. In this study, we aim to investigate the accuracy of AI content detectors, estimate the change in utilization of AI, and determine characteristics associated with AI content in scientific abstracts submitted to the ASCO Annual Meetings from 2021 to 2023.

## METHODS

### Study Design and Data Source

This retrospective study used abstracts submitted and accepted for publication to ASCO Annual Meetings from 2021 to 2023, accessed through ASCO's Data Library,^17^ although abstracts withdrawn from publication by authors were not available in the data library and thus not included. Key characteristics were tabulated for each abstract including abstract track, venue of presentation, inclusion of clinical trial number, as well as the countries and regions of the institutions the first author was affiliated with. First author institution region was derived from listed country using World Bank region classification with adjacent regions combined into a single category for some regions because of small subgroup size. Abstract data originated from a database intended to populate abstracts on the ASCO submission portal, and text fields included HTML code to render typographic changes and data tables. HTML tags, data tables, and special characters were removed using regular expressions to create plain text that can be parsed by AI content detectors. This study was determined to be exempt from review by the University of Chicago institutional review board.

### AI Content Detector Selection and Score Calculation

To identify AI content detectors, we conducted a search up to December 31, 2023, in PubMed Central for the terms language AND detector AND LLM OR GPT, resulting in 188 results, eight of which were studies describing the use of detectors to identify AI-written text. Of the detectors listed in these studies, we selected three AI content detectors that had a publicly available application programming interface (API) to allow for automatic processing of a large volume of abstracts, and which did not have a prohibitive cap on the amount of text that could be screened or the rate at which text could be screened (Appendix Table A1). The three detectors selected were GPTZero (version 2), Originality.ai (AI Scan), and Sapling. The raw text of each abstract was sequentially fed into each detector using the associated API to yield a numeric result (ranging from 0 to 1) representing the predicted likelihood of AI content in the abstract. To determine concordance between different detectors, we computed the Spearman correlation and agreement between the predictions for each pair of detectors.

### Accuracy of Detection With True-Positive and True-Negative Abstracts

As these detectors are not tuned specifically to identify AI content in oncology scientific abstracts, we evaluated performance in AI-generated and human-written abstracts. Optimal thresholds for each detector were selected by maximizing a weighted Youden's index for AI-generated and human-written abstracts, with specificity weighted by a factor of two to minimize false positives.^18^ We used 100 randomly selected abstracts from the years 2018-2019 to serve as putatively human-written true-negative controls, given these abstracts were created before the advent of modern LLMs. We also compiled 200 true-positive abstracts, with 100 each generated from OpenAI's GPT-3.5 and GPT-4 models, using the titles from the human-written abstracts and the prompt, “Please write an abstract for a paper titled <title> including sections for Background, Methods, and Results.”

As LLMs may be used for AI generation of small portions of abstract text rather than fabricating the scientific results of the study, we created a set of 200 mixed abstracts by combining the corresponding AI-written Background sections with the Methods, Results, and Conclusions sections from the human-written abstracts. Although the primary analysis was conducted using thresholds that best distinguish human-written from purely AI-generated abstracts, we applied a similar approach to identify the best threshold for human-written versus mixed AI/human abstracts. Finally, as modern translation software programs use LLM methodology, we assessed the impact of translation of human-written abstracts to Spanish and back to English with Google Translate. To assess the performance of detectors in these settings, we computed the area under the receiver operating curve (AUROC), area under the precision recall curve, and Brier score for comparison of the putative human-written abstracts to fully AI-generated, mixed human/AI, and translated abstracts.

### Characterization of AI Content Detector Predictions

To illustrate the changing distribution of AI content detection scores, we plotted the histogram of content detection scores in 2023 versus 2021-2022, using the Kolmogorov-Smirnov (KS) test to statistically test the difference in distribution of scores across detectors. Multivariable logistic regression models were fit for three AI content detection scores dichotomized at the optimal thresholds described above. Main effects for year, abstract track, abstract prefix, clinical trial identifier, and first author institution region were included in all multivariable models. Likelihood ratio tests were performed to guide selection of interaction terms of each main effect with year. To determine specific content that may trigger high AI content detection scores, we also conducted an analysis of words associated with dichotomized AI content detection scores for each detector. Abstracts were categorized as containing or not containing each word present in the entire corpus of text, with the exclusion of words with fewer than five characters, non-numeric characters, or appearing in fewer than 10 abstracts. Further univariable logistic regression models were fit to assess the association of individual words with high AI content detection scores.

### Statistical Analysis

Statistical analysis was done using both RStudio with R version 4.2.2 for summary of abstract characteristics and association of these characteristics with likelihood of AI content, as well as Python 3.9, scikit-learn 1.2.1, statsmodels 0.14.1, matplotlib 3.4.3, and seaborn 0.13.2 for plotting distributions of AI content scores and associations of individual words with AI content detection. All statistical testing was two-sided and performed at the α = .05 significance level. False discovery rate (FDR) correction with the Benjamini/Hochberg procedure was performed for univariable odds ratios (ORs) for individual words associated with high/low likelihood of AI content, given the large number of words assessed.

## RESULTS

**FIG 1.**
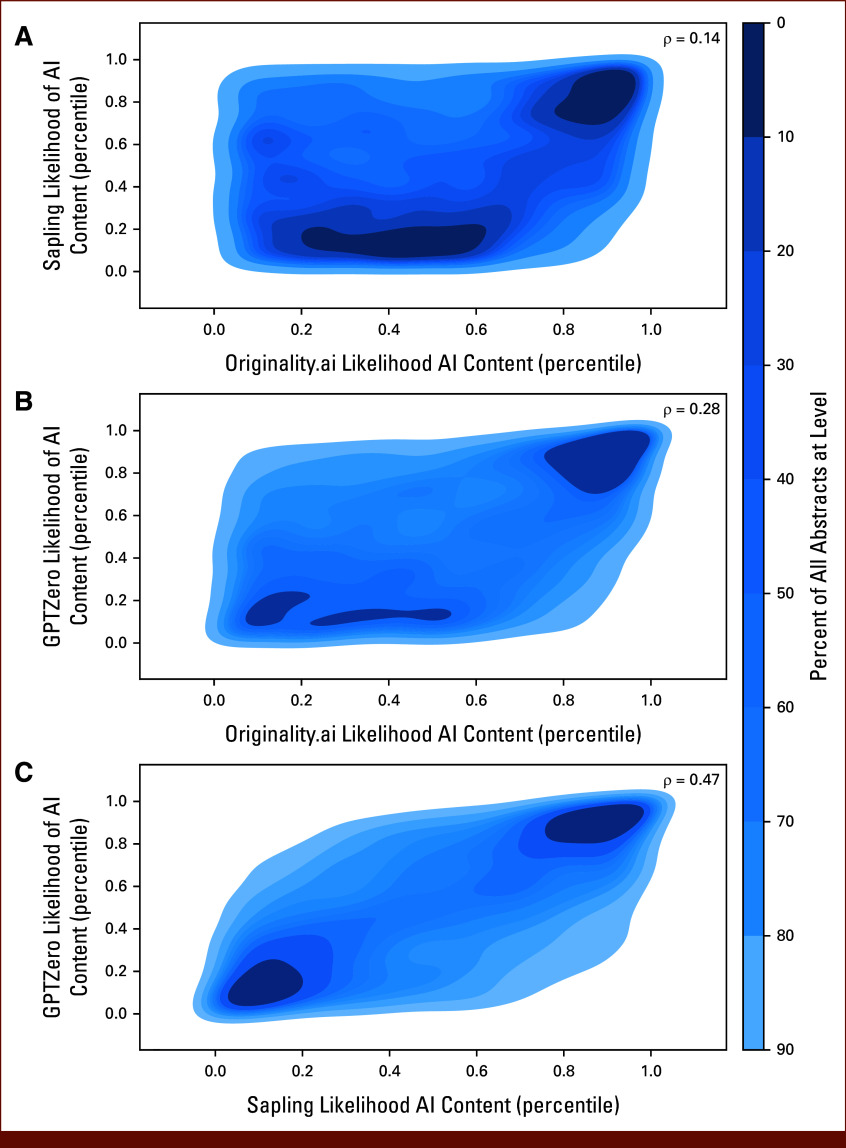
Correlation between outputs across pairs of AI content detectors: (A) Originality.ai and Sapling, (B) Originality.ai and GPTZero, and (C) Sapling and GPTZero. Shown is the kernel distribution estimation for the quantile-normalized output for each pair of detectors across all 15,553 abstracts. Each plotted contour represents 10% of all abstracts, ranging from high density (dark blue) to low density (light blue). The Spearman rank correlation coefficient (ρ) for the association between detector outputs is listed for each plot. AI, artificial intelligence.

**FIG A1.**
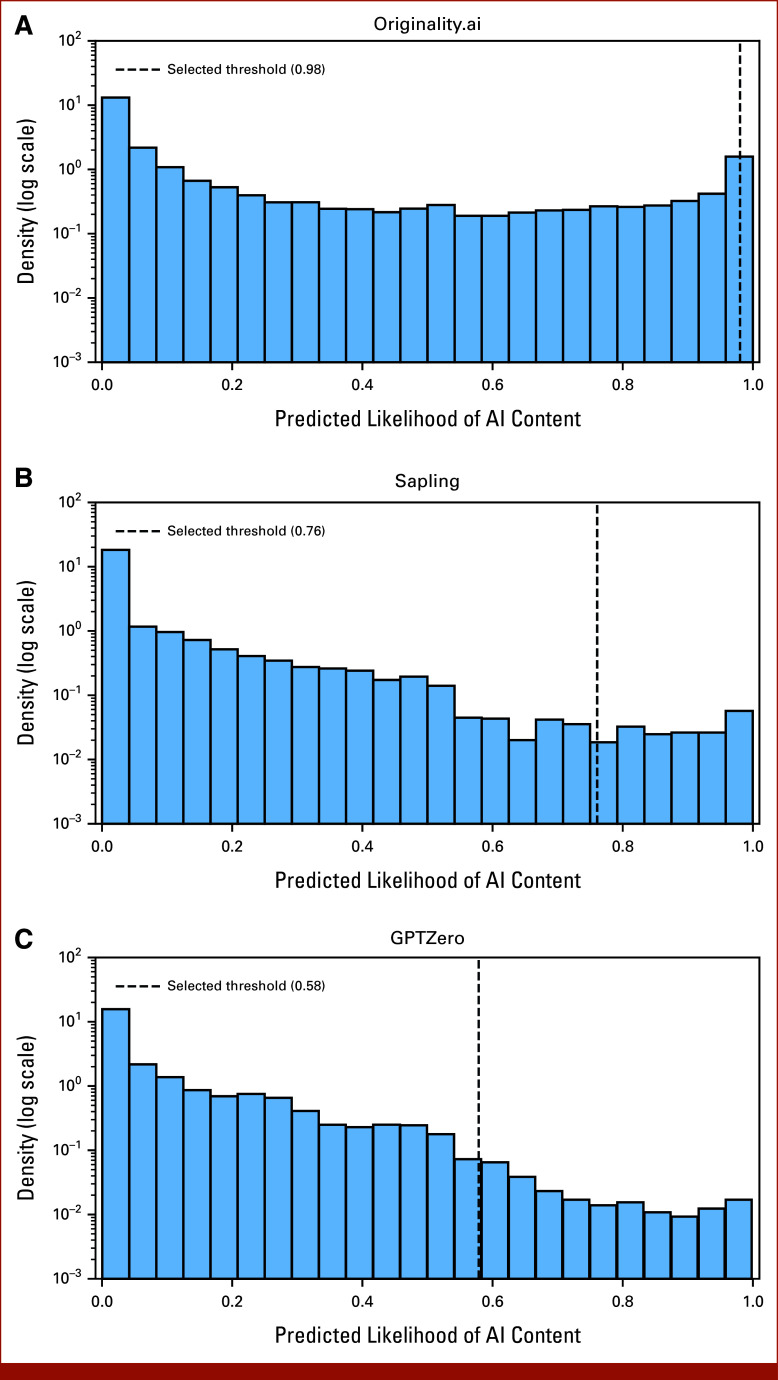
Distribution of raw detector outputs of three AI content detectors: (A) Originality.ai, (B) Sapling, and (C) GPTZero. Shown is the density of the raw output of the three detectors for the 15,553 abstracts included from 2021 to 2023. Each detector outputs an estimated probability of the text containing AI-generated content but, as illustrated, these probabilities drastically differ between each detector. Whereas no abstract received a probability of over 50% per the AK detector, 8.9% of abstracts were given a probability of over 90% per the Originality.ai detector. As these detectors were not trained specifically to identify content in scientific abstracts, these raw probabilities may not be accurate, and thus, we used an optimal threshold that best discriminated between a set of human-written and AI-generated abstracts (illustrated with a vertical dotted line) as a cutoff for higher likelihood of AI content in this study. AI, artificial intelligence.

A total of 15,553 ASCO Scientific Abstracts were extracted for analysis (Table 1). The volume of published abstracts increased from 2021 (n = 4,595; 29.5%) to 2022 (n = 5,198; 33.4%) and 2023 (n = 5,760; 37.0%). Most abstracts (n = 10,243; 65.9%) were published in cancer-specific tracks, did not include a clinical trial identifier (n = 10,226; 65.7%), and were submitted by authors at institutions in North America (n = 8,899; 57.2%). There was notable heterogeneity in predicted likelihood scores of AI content across detectors (Appendix Fig A1). GPTZero and Sapling demonstrated similar trends, with most abstracts receiving a very low likelihood of AI content. Originality yielded higher AI-generated likelihood scores than any other detector. To evaluate the concordance of predictions across detectors, we calculated the Spearman correlation between outputs of each pair of detectors (Appendix Table A2; Fig 1) and found that Sapling and GPTZero exhibited the strongest correlation (ρ = 0.471; 95% CI, 0.458 to 0.483; *P* < .001).

**TABLE 1.** Characteristics of ASCO Annual Meeting Abstracts and Authors, 2021-2023

Characteristic	2021 (n = 4,595), No. (%)	2022 (n = 5,198), No. (%)	2023 (n = 5,760), No. (%)	Overall (N = 15,553), No. (%)
Abstract track				
Cancer-specific tracks	3,036 (66.1)	3,460 (66.6)	3,747 (65.1)	10,243 (65.9)
Care delivery, quality, and health services	612 (13.3)	654 (12.6)	796 (13.8)	2,062 (13.3)
Developmental therapeutics	494 (10.8)	578 (11.1)	643 (11.2)	1,715 (11.0)
Medical education and professional development	61 (1.3)	78 (1.5)	53 (0.9)	192 (1.2)
Prevention, risk reduction, and genetics	140 (3.0)	143 (2.8)	185 (3.2)	468 (3.0)
Symptom science and palliative care	252 (5.5)	285 (5.5)	336 (5.8)	873 (5.6)
Presentation venue				
In person	2,458 (53.5)	2,854 (54.9)	2,927 (50.8)	8,239 (53.0)
Online only	2,137 (46.5)	2,344 (45.1)	2,833 (49.2)	7,314 (47.0)
Clinical trial ID				
Not present	3,057 (66.5)	3,366 (64.8)	3,803 (66.0)	10,226 (65.7)
Present	1,538 (33.5)	1,832 (35.2)	1,957 (34.0)	5,327 (34.3)
First author institution region				
North America	2,598 (56.5)	2,939 (56.5)	3,362 (58.4)	8,899 (57.2)
East Asia and Pacific	925 (20.1)	1,094 (21.0)	1,231 (21.4)	3,250 (20.9)
Europe and Central Asia	838 (18.2)	899 (17.3)	854 (14.8)	2,591 (16.7)
Latin America and Caribbean	98 (2.1)	100 (1.9)	134 (2.3)	332 (2.1)
Middle East, North Africa, and Sub-Saharan Africa	65 (1.4)	74 (1.4)	89 (1.5)	228 (1.5)
South Asia	71 (1.5)	92 (1.8)	90 (1.6)	253 (1.6)

**FIG 2.**
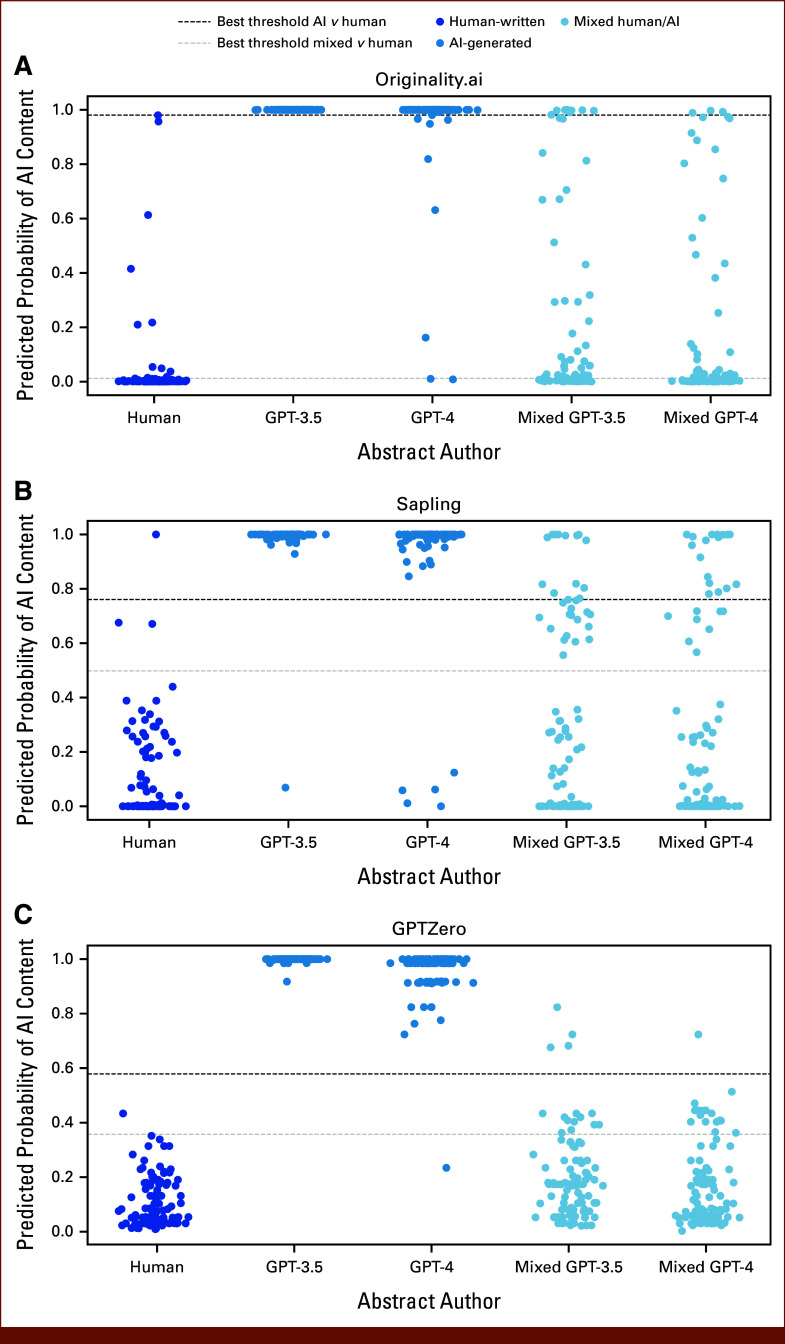
Accuracy of AI content detectors in classifying human-written and AI-generated content: (A) Originality.ai, (B) Sapling, and (C) GPTZero. One hundred ASCO abstracts from 2018 to 2019, before the advent of modern large language models, were used as human-written negative controls. One hundred abstracts were generated each by OpenAI's GPT-3.5 and GPT-4 models as positive controls, using the titles from the human-written abstracts as part of the prompt for generation. As authors may use AI to write small sections of scientific work, we also created mixed abstracts that merged the Background section from the AI-generated abstracts with human-written Methods, Results, and Conclusion sections. As illustrated, GPTZero had the best discrimination of the pure AI-generated abstracts at an optimal threshold selected with Youden's index, identifying 99.5% of AI-written abstracts with no false positives among human-written text. AI, artificial intelligence.

To determine thresholds for AI content detection in this data set, a weighted Youden's index was used to optimize discrimination of human-written abstracts from GPT-3.5–/GPT-4–generated abstracts (Fig 2; Appendix Table A3). With optimal thresholds, 99.5%, 96%, and 97% of GPT-3–/GPT-4–generated abstracts were identified by GPTZero, Originality, and Sapling, respectively, and no sampled abstracts from 2018 to 2019 were classified as AI generated by the GPTZero and Originality detectors. Only a single abstract from 2018 to 2019 was classified as AI-generated by Sapling using these optimal thresholds. Of note, translation of abstracts to Spanish language and back to English led to a 33% classification as AI content by Originality with the chosen threshold, but only 3% positive rate with Sapling and no positive classifications with GPTZero. When using optimal thresholds to identify mixed human/AI abstracts, 47.5%, 27.5%, and 15.5% of such abstracts were detected by Originality, Sapling, and GPTZero, with false-positive rates of 11%, 3%, and 1%, respectively, for human-written abstracts from 2018 to 2019. The high accuracy of detectors for fully AI-generated content is reflected in AUROC scores (ranging 0.973 with Sapling to 0.999 with GPTZero for identifying GPT-4–generated abstracts), whereas accuracy for partial AI content was modest (AUROC ranging from 0.596 with GPTZero to 0.706 with Originality for identifying mixed human/GPT-4–generated abstracts; Appendix Table A4).

**FIG 3.**
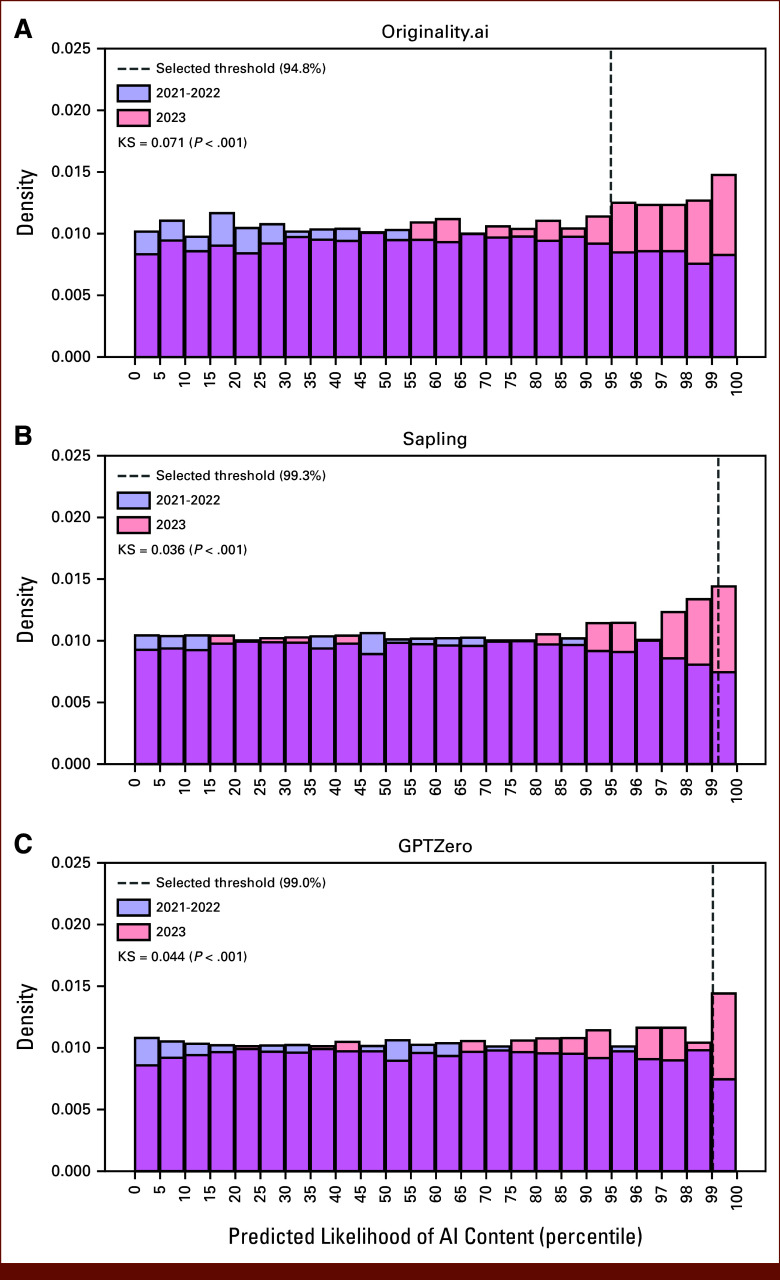
Distribution of normalized outputs of three AI content detectors for 2023 versus previous years: (A) Originality.ai, (B) Sapling, and (C) GPTZero. Shown is the distribution of quantile-normalized likelihood of AI generation output of the three detectors for the 5,760 abstracts in 2023 versus the 9,763 abstracts from 2021 and 2022. Abstracts from 2023 make up a greater proportion of high AI content scores across all detectors. This difference in distribution between 2023 and previous years was significant per the KS test, with test statistic and *P* value for each detector illustrated above. The quantile of abstracts classified as likely containing AI content with the optimal threshold for each detector is illustrated. Quantiles are shown at 5% intervals up to 95% and 1% intervals thereafter to better illustrate changes in distribution of abstracts with the highest AI content scores across the three detectors. AI, artificial intelligence; KS, Kolmogorov-Smirnov.

**FIG A2.**
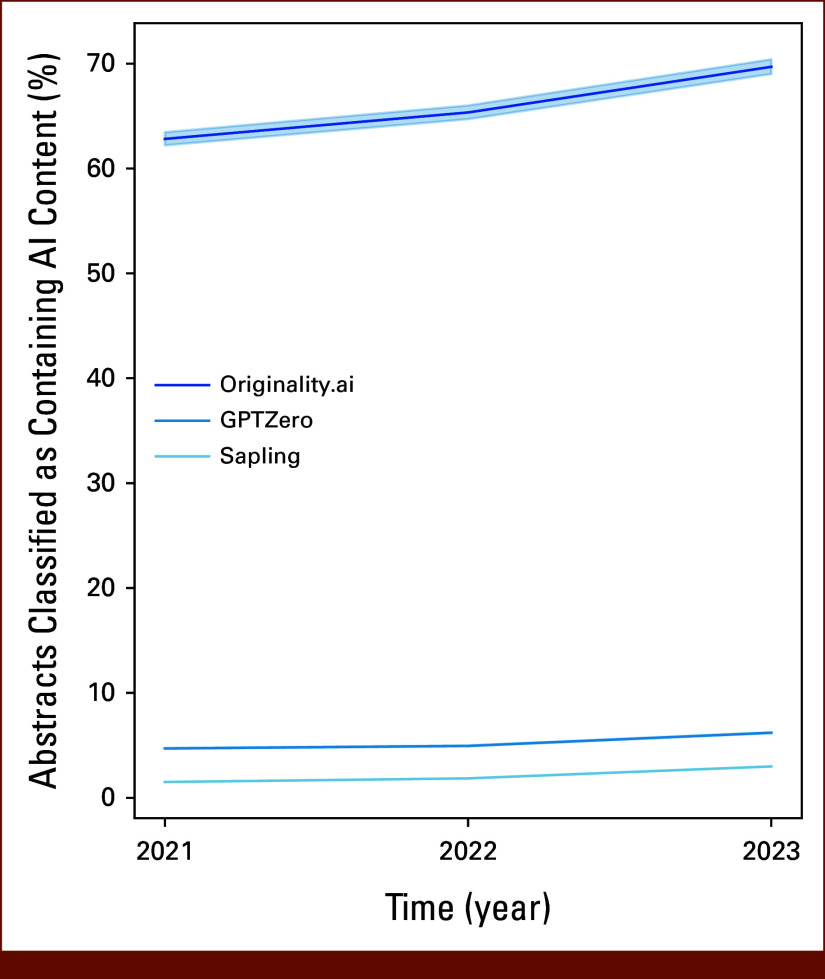
Percentage of abstracts classified as AI-generated using selected thresholds by year. Shown is the percentage of ASCO abstracts classified as AI-generated by the three detectors from years 2021 to 2023 using the identified optimal thresholds for distinguishing AI-generated versus human-written text. Shaded area around each bar represents the 95% CIs estimated using the Clopper-Pearson exact method, although CIs are difficult to visualize for the GPTZero and Sapling detectors because of the high sample size. AI, artificial intelligence.

In the full 15,553 abstract data set, a greater proportion of abstracts from 2023 compared with 2021 met the optimal threshold for high AI content score—with increases ranging from 218% with Originality (increase from 177 to 386 abstracts) to 295% with Sapling (increase from 21 to 62 abstracts; Appendix Table A5 and Fig A2). The distribution of scores from AI content detectors differed in 2023 versus earlier years (Fig 3), with a higher representation of 2023 abstracts in the upper end of AI content scores. This difference in distribution was consistently significant across detectors (KS *P* < .01 for all detectors). These findings remained significant in a fully adjusted logistic regression—compared with 2021, abstracts submitted from 2023 (but not 2022) had a significantly higher odds of AI content detection across all detectors (aOR range, 1.79; 95% CI, 1.50 to 2.16 with Originality to 2.37; 95% CI, 1.47 to 3.98 with Sapling; Table 2). Abstracts triaged to online-only presentation were also associated with a higher odds of AI content (aOR range, 2.36; 95% CI, 1.39 to 4.15 with Sapling to 3.79; 95% CI, 2.99 to 4.86 with Originality). Abstracts with a clinical trial ID included were associated with lower odds of AI content (aOR range, 0.02; 95% CI, 0.00 to 0.11 with GPTZero to 0.40; 95% CI, 0.31 to 0.52 with Originality). There was a trend toward increased AI content in the “Prevention, Risk Reduction, and Genetics,” but this was not significant across all detectors.

**TABLE 2.** Unadjusted Logistic Regression Models for Three AI Content Detectors

Variable	Sapling OR (95% CI)	GPTZero OR (95% CI)	Originality.ai OR (95% CI)
Year			
2021	–Ref	–Ref	–Ref
2022	1.39 (0.81 to 2.44)	1.02 (0.64 to 1.63)	1.20 (0.98 to 1.46)
2023	2.37 (1.47 to 3.98)	1.92 (1.29 to 2.93)	1.79 (1.50 to 2.16)
Abstract track			
Cancer-specific tracks	–Ref	–Ref	–Ref
Care delivery, quality, and health services	2.94 (1.54 to 5.44)	1.16 (0.58 to 2.11)	0.89 (0.64 to 1.20)
Developmental therapeutics	1.58 (0.63 to 3.45)	0.24 (0.04 to 0.76)	0.48 (0.30 to 0.72)
Medical education and professional development	8.59 (2.00 to 25.43)	NE^a^	0.25 (0.01 to 1.14)
Prevention, risk reduction, and genetics	4.80 (1.77 to 11.06)	4.77 (2.32 to 9.00)	1.57 (0.94 to 2.47)
Symptom science and palliative care	1.72 (0.51 to 4.46)	1.14 (0.39 to 2.62)	0.91 (0.56 to 1.39)
Presentation venue			
In person	–Ref	–Ref	–Ref
Online only	2.36 (1.39 to 4.15)	2.89 (1.78 to 4.88)	3.79 (2.99 to 4.86)
Clinical trial ID			
Not present	–Ref	–Ref	–Ref
Present	0.13 (0.04 to 0.32)	0.02 (0.00 to 0.11)	0.40 (0.31 to 0.52)
First author institution region			
North America	–Ref	–Ref	–Ref
East Asia and Pacific	0.94 (0.46 to 1.76)	0.84 (0.46 to 1.44)	2.69 (2.14 to 3.38)
Europe and Central Asia	0.45 (0.13 to 1.12)	0.07 (0.00 to 0.34)	0.51 (0.32 to 0.78)
Latin America and Caribbean	4.46 (1.66 to 10.05)	2.98 (1.13 to 6.55)	2.34 (1.29 to 3.96)
Middle East, North Africa, and Sub-Saharan Africa	4.47 (1.32 to 11.51)	2.22 (0.53 to 6.19)	2.62 (1.29 to 4.81)
South Asia	1.07 (0.06 to 5.03)	0.72 (0.04 to 3.31)	2.06 (0.95 to 3.96)

Abbreviations: AI, artificial intelligence; NE, not estimable; OR, odds ratio; Ref, reference.

a Odds ratio not estimable; no abstracts in this category met the specified threshold.

Similarly, there were increases in abstracts meeting the optimal thresholds for detecting mixed AI/human content from 2021 to 2023, with increases ranging from 39% with Originality (increase from 2,887 to 4,015 abstracts) to 249% with GPTZero (increase from 69 to 172). Of note, the majority of abstracts from all years met the threshold for containing mixed AI/human content with Originality, suggesting the false-positive rate may be higher in contemporary abstracts with the selected threshold. Nonetheless, abstracts from 2023 were consistently associated with a higher odds of mixed AI/human content across all detectors on a fully adjusted logistic regression (aOR range, 1.33; 95% CI, 1.11 to 1.58 with GPTZero to 2.00; 95% CI, 1.51 to 2.67 with Sapling), with similar associations seen with AI content and other abstract characteristics (Appendix Table A6).

**FIG 4.**
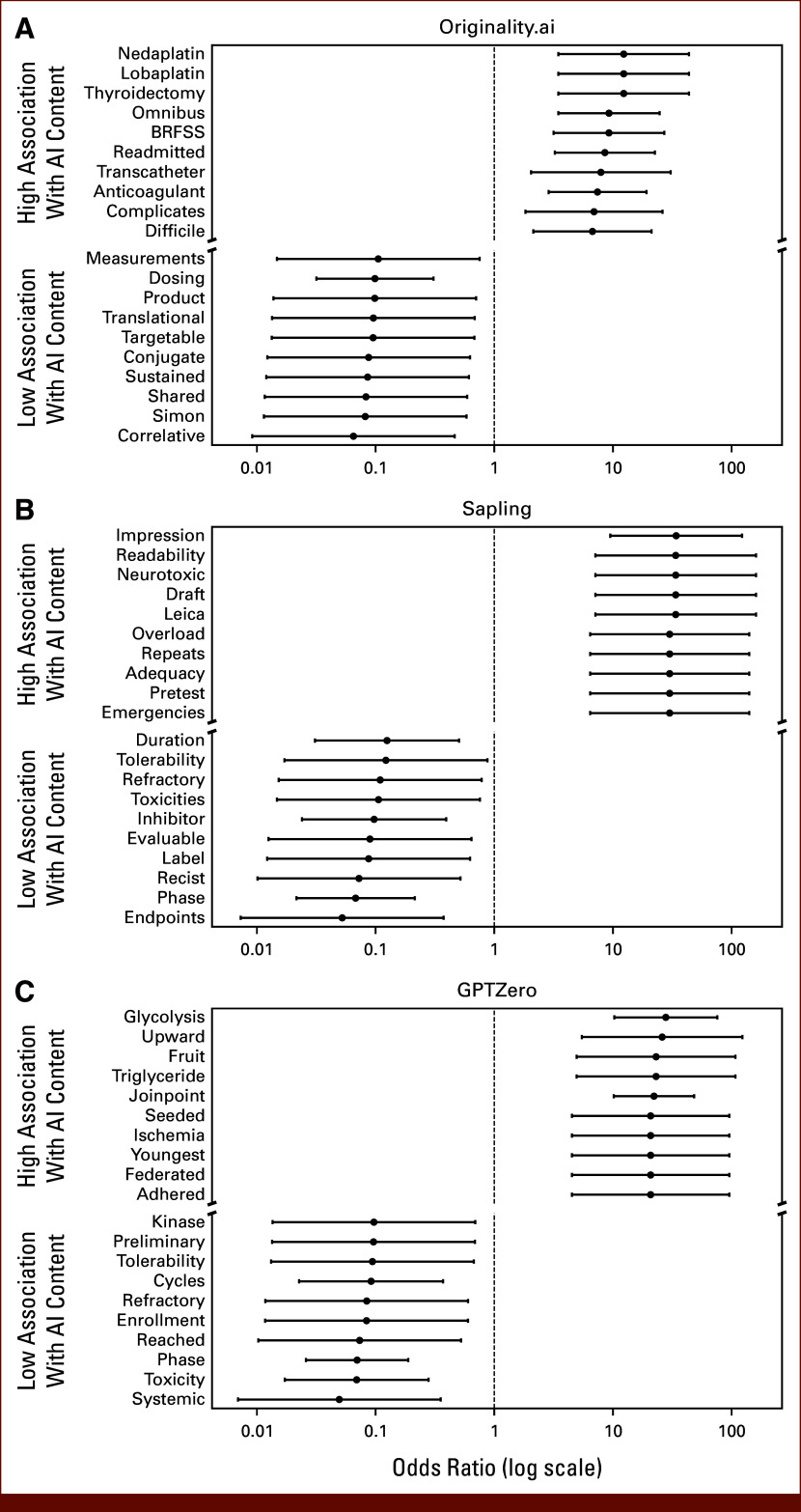
Association of individual words with low and high likelihood of AI content across detectors: (A) Originality.ai, (B) Sapling, and (C) GPTZero. Univariable odds ratios and CIs for the association of each word with high probability AI content (using optimal thresholds that best discriminated true human-written/AI-generated abstracts) were computed for each AI content detector. Words with fewer than five characters or appearing fewer than 10 times across all abstracts were excluded. The words with the 10 strongest positive and negative associations with AI content are plotted for each detector. Words associated with geographic regions were excluded from this plot. AI, artificial intelligence.

There were 8,097 unique non-numeric words with over four characters and contained in at least 10 abstracts each. When evaluating the top 20 words associated with the highest and lowest ORs for AI content across detectors (Fig 4), words associated with clinical treatment (“cycles,” “dosing”), adverse events (“toxicities,” “tolerability”), or trial design (“simon,” “phase”) were generally associated with lower likelihood of AI content. Across all detectors, words such as “trial” (OR range, 0.10 [95% CI, 0.05 to 0.19]; FDR-corrected *P* < .001 with GPTZero to 0.41; 95% CI, 0.35 to 0.49; FDR-corrected *P* < .001 with Originality) and “phase” (OR range, 0.07 [95% CI, 0.02 to 0.21]; FDR-corrected *P* < .001 for Sapling, to 0.31; 95% CI, 0.25 to 0.39; FDR-corrected *P* = .001 with Originality) were significantly associated with lower likelihood of AI content after FDR correction (Appendix Table A7).

## DISCUSSION

To our knowledge, this study represents the largest evaluation of multiple state-of-the-art AI content detectors across a single body of contemporary scientific abstracts. Our results demonstrate a significant increase in AI content detection in the year 2023, which coincides with the rise in popularity of LLM chatbots such as ChatGPT and reinforces studies describing AI proficiency at scientific content generation. We demonstrate that AI detection signal was associated with abstracts selected for online-only presentation, as well as the absence of an associated clinical trial number. Although there were trends toward association with AI content on the basis of the track of abstract submission and geographic region of submission, these were not consistently significant across detectors.

Several other studies have compared AI content detection in scientific abstracts on smaller scales or with fewer detectors. In an earlier report of 50 real and 50 ChatGPT-generated scientific abstracts, Gao et al^14^ demonstrated that a GPT-2 content detector could better distinguish the generated scientific abstracts than human reviewers, but there was no threshold that perfectly distinguished the AI-generated abstracts. Rashidi et al^19^ describe the performance of a GPT-2 content detector in 14,440 abstracts from top scientific journals, demonstrating positive signal in years before the advent of LLMs indicative of false-positive results. Converse to other studies, Rashidi et al^19^ found a decrease in AI detection signal in more recent years, but there was no granular comparison of 2023 (where LLM uptake has increased) to previous years. However, this older GPT-2 detector was not designed to distinguish text from modern LLMs compared with most of the detectors evaluated in our study.

The accuracy of tools evaluated in this study for detection of AI-generated medical or scientific tests has been reported in a small number of studies. Miller et al^20^ demonstrated a sensitivity of 100% and a specificity of 95% for Originality in characterizing a sample of 60 human-written and AI-generated abstracts. This study also evaluated a selection of 390 abstracts from randomized clinical trials published in 2020-2023 with Originality, demonstrating an increase in AI content detection in 2023.^20^ GPTZero has also been evaluated for the detection of AI content in smaller-scale studies of scientific text, with sensitivities ranging from 27% to 93%, and specificities ranging from 80% to 90%,^21,22^ whereas no studies were found on a literature search reporting the accuracy of Sapling in medical text. The reported accuracies are difficult to compare across studies, as predictions are dependent on the AI model used for content generation, size of the text, and context of text generation. In our work, we found that all detectors had sensitivity and specificity of over 95% for AI content. GPTZero achieved the highest AUROC/lowest Brier score for all human-written/fully AI-generated comparisons, perhaps making this the most appealing screening tool for completely AI-generated content. Detectors had limited ability to distinguish mixed AI-generated/human-written content, and others have reported that AI content detection is less accurate with smaller lengths of text.^19^ In reality, most authors would likely use AI to generate only small sections of abstracts rather than relying on fully AI-generated text, and more accurate tools to identify AI content on a sentence or phrase level, as opposed to global text classification, are needed.

Although we found that AI content detection may differ by the geographic region of authors, great care must be taken to ensure AI detection does not perpetuate disparities in scientific literature publication, recognizing that AI detection does not imply poor science or fraudulent findings, and false positives are not uncommon. One study compared predictions from GPT detectors for essays written by Chinese students for an English proficiency examination with those written by US eighth-grade students, demonstrating a high rate of false-positive detection in the former essays.^23^ Furthermore, we demonstrate that translation software, which may use LLM foundations, might trigger some AI content detectors. The ethical use of LLMs to improve the quality and clarity of text from non-English speakers has also been advocated, as these tools may improve the dissemination of important scientific findings to the global research community.^24^ As the utilization of AI detection tools increases, it will be important to ensure equitable application to authors from all global regions—perhaps through the development of region-specific cutoffs for these tools and to develop policies to guide use of AI, which would ideally be consistent for medical journals and abstract submissions.

The usage of AI in scientific writing comes with significant ethical concerns such as plagiarism and attribution of authorship. Usage of AI-generated content may lead to unintentional plagiarism when content sources are not provided along with the generated texts, or can generate fictitious/hallucinated content—particularly citations to nonexistent articles.^25^ LLMs may also inadvertently perpetuate the bias within the large web-based data sets they are trained on, including bias regarding sex, race, ethnicity, and disability status.^26^ Well-defined guidelines are needed to regulate ethical usage of AI in academic writing. Although LLMs might not be able to independently produce comprehensive and factual scientific writing, they could provide help in editing and improve the narrative of academic writing. GPT-4 was found to be able to assist researchers in revising medical abstracts and enhance the quality of the original writing.^27^ Medical domain–specific LLMs have also been created by fine-tuning open-source LLMs on biomedical academic papers to improve performance on benchmark tests.^28^ The academic writing ability of such models has yet to be explored. Even so, authors choosing to use AI to create content for scientific writing of abstracts and other publications should be held accountable for all aspects of the submission, requiring their due diligence to ensure factual and accurate representation of content with no misleading or inaccurate information. LLMs and AI can be used as tools for facilitation of scientific writing but they do not absolve authors of the full responsibility of the accuracy of the products they submit for publication.

The primary limitation of this study is the lack of a ground truth for AI content detection. Unfortunately, outside of study authors admitting to the use of AI, or content with clearly generated phrases such as “As a LLM trained by OpenAI,” we suspect that identification of generated text will never reach perfect accuracy and as demonstrated here, commonly used detectors do not show high agreement in likelihood scores. Therefore, AI content detectors should not be used as the sole means to assess AI content on scientific writing but could be used as a screening tool to indicate that the presented content requires additional scrutiny from reviewers.

In conclusion, there is evidence of increased AI content within oncology scientific abstracts in 2023 compared with previous years, associated with online-only publications and lack of inclusion of clinical trial registration number. Contemporary AI content detectors have the potential to be tuned to distinguish AI content and may be useful as a supplementary screening tool for scientific review. However, there is variability in detection scores from commonly used detectors, suggesting the need for careful consideration of the choice of detector and an understanding of operating characteristics for detectors for specific types of publications.

## Data Availability

ASCO scientific abstract data sets can be made available through completion of a data request through the ASCO Data Library https://society.asco.org/research-data/asco-data-library. All other data collected for this study are available from the authors upon reasonable request. Code used to query the API for AI content detectors and conduct scientific analysis is provided at https://github.com/anranli0/AbstractAudit.

## APPENDIX

**TABLE A1.** List of AI Content Detectors Considered in this Study, and Reasons for Exclusion

Detector	Selection/Exclusion	Link
GPTZero	Selected	gptzero.me
Originality.ai	Selected	originality.ai
Sapling	Selected	sapling.ai
Kashyap	Excluded because of limited performance in distinguishing human-written *v* generated abstracts	rapidapi.com/arshk102001/api/ai-content-detector2
Copyleaks AI content detector	Not enough usage allowed to process entire data set	copyleaks.com/ai-content-detector
Writer	Not enough usage allowed to process entire data set	writer.com/ai-content-detector
AI Content at Scale	No API access at the time of analysis	contentatscale.ai
OpenAI RoBERTa GPT-2 detector	No API available without custom configuration on Hugging Face	openai-openai-detector.hf.space
ZeroGPT	Cost-prohibitive	zerogpt.com
Undetectable.ai	Limited words per month on noncommercial subscriptions	undetectable.ai

Abbreviations: AI, artificial intelligence; API, application programming interface.

**TABLE A2.** Correlation and Agreement Between Predictions From Pairs of Detectors

Detector 1	Detector 2	Spearman ρ (95% CI)	*P*	Agreement, No. (%)	Positive for Both Detectors, No. (%)
Originality.ai	Sapling	0.143 (0.128 to 0.159)	5.7 × 10^–72^	14,672 (94.6)	36 (0.23)
Originality.ai	GPTZero	0.284 (0.270 to 0.298)	<1 × 10^–100^	14,744 (94.8)	71 (0.46)
Sapling	GPTZero	0.471 (0.458 to 0.483)	<1 × 10^–100^	15,306 (98.5)	19 (0.12)

NOTE. Analysis was conducted on all 15,553 abstracts included in this study.

**TABLE A3.** Performance of Different Thresholds for Each AI Content Detector in Classifying Human-Written, AI-Generated, Mixed, and Translated Abstracts

Detector	Human-Written (n = 100)	Mixed, GPT-3.5 (n = 100)	Mixed, GPT-4 (n = 100)	Generated, GPT-3.5 (n = 100)	Generated, GPT-4 (n = 100)	Translated (n = 100)
Abstracts per category classified as high likelihood of AI content with specified threshold						
GPTZero	0	4	1	100	99	0
Originality.ai	0	9	3	100	92	33
Sapling	1	13	17	99	95	3
Abstracts per category classified as high likelihood of mixed AI/human content with specified threshold						
GPTZero	1	15	16	100	99	5
Originality.ai	11	51	44	100	98	82
Sapling	3	30	25	99	95	7

Abbreviation: AI, artificial intelligence.

**TABLE A4.** Performance Characteristics of AI Content Detectors in Distinguishing AI-Generated Text From Human-Written Abstracts

Detector	Comparison	AUROC	AUPRC	Brier Score
Originality.ai	GPT-3.5 *v* human-written	**1.000**	**1.000**	0.013
	GPT-4 *v* human-written	0.995	0.996	0.027
	Mixed human/GPT-3.5 *v* human-written	**0.782**	**0.775**	0.400
	Mixed human/GPT-4 *v* human-written	**0.706**	**0.703**	0.426
	Translated *v* human-written	**0.912**	**0.921**	**0.199**
Sapling	GPT-3.5 *v* human-written	0.991	0.990	0.024
	GPT-4 *v* human-written	0.973	0.975	0.043
	Mixed human/GPT-3.5 *v* human-written	0.617	0.671	**0.338**
	Mixed human/GPT-4 *v* human-written	0.606	0.655	**0.359**
	Translated *v* human-written	0.609	0.595	0.397
GPTZero	GPT-3.5 *v* human-written	**1.000**	**1.000**	**0.010**
	GPT-4 *v* human-written	**0.999**	**0.999**	**0.016**
	Mixed human/GPT-3.5 *v* human-written	0.684	0.692	0.346
	Mixed human/GPT-4 *v* human-written	0.596	0.636	0.372
	Translated *v* human-written	0.585	0.586	0.385
Kashyap^a^	GPT-3.5 *v* human-written	0.970	0.970	0.477
	GPT-4 *v* human-written	0.880	0.826	0.498
	Mixed human/GPT-3.5 *v* human-written	0.670	0.701	0.499
	Mixed human/GPT-4 *v* human-written	0.640	0.702	0.499

NOTE. Each comparison represents performance metrics for detecting 100 human-written versus 100 generated, mixed, or translated abstracts. However, the Kashyap detector underwent preliminary assessment with 10 human-written versus 10 generated or mixed abstracts with performance as listed, but because of inferior performance compared with other detectors was excluded for future analysis. Bold text indicates the model achieved the best performance for the specified comparison as measured by the listed metric (ie, highest AUROC or AUPRC, or lowest Brier Score).

Abbreviations: AI, artificial intelligence; AUPRC, area under the precision recall curve; AUROC, area under the receiver operating characteristic curve.

a Detector was run on a preliminary cohort with 10 abstracts in each category, but further analysis was not performed because of lower accuracy compared with other detectors.

**TABLE A5.** Distribution of Predictions From AI Content Detectors by Year

Detector	KS Stat, 2021-2022 *v* 2023	*P*	Abstracts Predicted to Have AI Content by Year, No. (%)			Abstracts Predicted to Have Mixed AI/Human Content by Year, No. (%)		
			2021 (n = 4,595)	2022 (n = 5,198)	2023 (n = 5,760)	2021 (n = 4,595)	2022 (n = 5,198)	2023 (n = 5,760)
Originality.ai	0.071	1.613 × 10^-16^	177 (3.9)	238 (4.6)	386 (6.7)	2,887 (62.8)	3,397 (65.4)	4,015 (69.7)
Sapling	0.036	1.418 × 10^-4^	21 (0.5)	33 (0.6)	62 (1.1)	216 (4.7)	257 (4.9)	357 (6.2)
GPTZero	0.044	1.344 × 10^-6^	33 (0.7)	38 (0.7)	79 (1.4)	69 (1.5)	96 (1.8)	172 (3.0)

Abbreviations: AI, artificial intelligence; KS, Kolmogorov-Smirnov.

**TABLE A6.** Adjusted Logistic Regression Models for Three AI Content Detectors, Using Thresholds for Detecting Mixed AI/Human Content

Variable	Sapling aOR (95% CI)	GPTZero aOR (95% CI)	Originality.ai aOR (95% CI)
Year			
2021	–Ref	–Ref	–Ref
2022	1.25 (0.91 to 1.71)	1.08 (0.90 to 1.31)	1.11 (1.02 to 1.21)
2023	2.00 (1.51 to 2.67)	1.33 (1.11 to 1.58)	1.36 (1.25 to 1.47)
Abstract track			
Cancer-specific tracks	–Ref	–Ref	–Ref
Care delivery, quality, and health services	2.68 (2.01 to 3.56)	0.88 (0.71 to 1.09)	0.64 (0.58 to 0.71)
Developmental therapeutics	1.86 (1.31 to 2.60)	1.02 (0.79 to 1.30)	0.69 (0.62 to 0.76)
Medical education and professional development	6.93 (3.93 to 11.64)	1.46 (0.83 to 2.41)	0.34 (0.26 to 0.46)
Prevention, risk reduction, and genetics	2.06 (1.13 to 3.48)	2.49 (1.84 to 3.32)	0.82 (0.67 to 1.00)
Symptom science and palliative care	1.62 (0.99 to 2.53)	1.68 (1.28 to 2.18)	0.61 (0.53 to 0.70)
Presentation venue			
In person	–Ref	–Ref	–Ref
Online only	1.42 (1.11 to 1.83)	1.56 (1.33 to 1.84)	0.96 (0.89 to 1.04)
Clinical trial ID			
Not present	–Ref	–Ref	–Ref
Present	0.57 (0.41 to 0.79)	0.11 (0.08 to 0.16)	0.97 (0.89 to 1.05)
First author institution region			
North America	–Ref	–Ref	–Ref
East Asia and Pacific	1.29 (0.96 to 1.72)	1.38 (1.15 to 1.66)	1.82 (1.66 to 2.01)
Europe and Central Asia	0.58 (0.38 to 0.86)	0.63 (0.49 to 0.80)	0.83 (0.75 to 0.91)
Latin America and Caribbean	1.40 (0.76 to 2.38)	1.75 (1.24 to 2.43)	1.53 (1.20 to 1.97)
Middle East, North Africa, and Sub-Saharan Africa	2.25 (1.22 to 3.84)	1.23 (0.74 to 1.92)	0.95 (0.72 to 1.25)
South Asia	0.31 (0.05 to 0.97)	0.16 (0.04 to 0.43)	0.96 (0.74 to 1.26)

Abbreviations: AI, artificial intelligence; aOR, adjusted odds ratios; Ref, reference.

**TABLE A7.** Association of Individual Words With Likelihood of AI Content, Assessed Through Univariable Logistic Regression

Detector	Word	OR (95% CI)	*P*	FDR Corrected *P*
Originality	Difficile	6.73 (2.14 to 21.17)	.001	.025
	Complicates	6.93 (1.83 to 26.17)	.004	.066
	Anticoagulant	7.41 (2.87 to 19.16)	<.001	.002
	Transcatheter	7.92 (2.04 to 30.68)	.003	.049
	Readmitted	8.56 (3.24 to 22.57)	<.001	<.001
	BRFSS	9.26 (3.16 to 27.16)	<.001	.002
	Omnibus	9.27 (3.47 to 24.76)	<.001	<.001
	Thyroidectomy	12.33 (3.47 to 43.8)	<.001	.004
	Lobaplatin	12.33 (3.47 to 43.8)	<.001	.004
	Nedaplatin	12.33 (3.47 to 43.8)	<.001	.004
	Correlative	0.07 (0.01 to 0.46)	.006	.088
	Simon	0.08 (0.01 to 0.58)	.013	.137
	Shared	0.08 (0.01 to 0.59)	.013	.141
	Sustained	0.09 (0.01 to 0.61)	.014	.148
	Conjugate	0.09 (0.01 to 0.62)	.015	.154
	Targetable	0.10 (0.01 to 0.68)	.019	.177
	Translational	0.10 (0.01 to 0.68)	.019	.179
	Product	0.10 (0.01 to 0.7)	.021	.187
	Dosing	0.10 (0.03 to 0.31)	<.001	.003
	Measurements	0.11 (0.01 to 0.75)	.025	.208
Sapling	Emergencies	30.07 (6.43 to 140.73)	<.001	<.001
	Pretest	30.07 (6.43 to 140.73)	<.001	<.001
	Adequacy	30.07 (6.43 to 140.73)	<.001	<.001
	Repeats	30.07 (6.43 to 140.73)	<.001	<.001
	Overload	30.07 (6.43 to 140.73)	<.001	<.001
	Leica	33.84 (7.11 to 161.08)	<.001	<.001
	Draft	33.84 (7.11 to 161.08)	<.001	<.001
	Neurotoxic	33.84 (7.11 to 161.08)	<.001	<.001
	Readability	33.84 (7.11 to 161.08)	<.001	<.001
	Impression	34.13 (9.50 to 122.57)	<.001	<.001
	Endpoints	0.05 (0.01 to 0.37)	.003	.03
	Phase	0.07 (0.02 to 0.21)	<.001	<.001
	Recist	0.07 (0.01 to 0.52)	.009	.062
	Label	0.09 (0.01 to 0.63)	.015	.084
	Evaluable	0.09 (0.01 to 0.64)	.016	.087
	Inhibitor	0.10 (0.02 to 0.39)	.001	.013
	Toxicities	0.11 (0.01 to 0.76)	.025	.110
	Refractory	0.11 (0.02 to 0.78)	.028	.118
	Tolerability	0.12 (0.02 to 0.88)	.036	.139
	Duration	0.12 (0.03 to 0.51)	.004	.031
GPTZero	Adhered	20.8 (4.52 to 95.76)	<.001	.003
	Federated	20.8 (4.52 to 95.76)	<.001	.003
	Youngest	20.8 (4.52 to 95.76)	<.001	.003
	Ischemia	20.8 (4.52 to 95.76)	<.001	.003
	Seeded	20.8 (4.52 to 95.76)	<.001	.003
	Joinpoint	22.19 (10.19 to 48.34)	<.001	<.001
	Triglyceride	23.11 (4.95 to 107.89)	<.001	.002
	Fruit	23.11 (4.95 to 107.89)	<.001	.002
	Upward	26.01 (5.48 to 123.50)	<.001	.001
	Glycolysis	27.92 (10.29 to 75.79)	<.001	<.001
	Systemic	0.05 (0.01 to 0.35)	.003	.035
	Toxicity	0.07 (0.02 to 0.28)	<.001	.004
	Phase	0.07 (0.03 to 0.19)	<.001	<.001
	Reached	0.07 (0.01 to 0.53)	.009	.084
	Enrollment	0.08 (0.01 to 0.6)	.014	.107
	Refractory	0.08 (0.01 to 0.6)	.014	.108
	Cycles	0.09 (0.02 to 0.37)	<.001	.013
	Tolerability	0.09 (0.01 to 0.67)	.019	.136
	Preliminary	0.10 (0.01 to 0.69)	.020	.137
	Kinase	0.10 (0.01 to 0.69)	.020	.137

NOTE. Words with top 10 highest and lowest ORs for each detector are shown.

Abbreviations: AI, artificial intelligence; FDR, false discovery rate; OR, odds ratio.
